# Performance of genetic risk factors in prediction of trichloroethylene induced hypersensitivity syndrome

**DOI:** 10.1038/srep12169

**Published:** 2015-07-20

**Authors:** Yufei Dai, Ying Chen, Hanlin Huang, Wei Zhou, Yong Niu, Mingrong Zhang, Ping Bin, Haiyan Dong, Qiang Jia, Jianxun Huang, Juan Yi, Qijun Liao, Haishan Li, Yanxia Teng, Dan Zang, Qingfeng Zhai, Huawei Duan, Juan Shen, Jiaxi He, Tao Meng, Yan Sha, Meili Shen, Meng Ye, Xiaowei Jia, Yingping Xiang, Huiping Huang, Qifeng Wu, Mingming Shi, Xianqing Huang, Huanming Yang, Longhai Luo, Sai Li, Lin Li, Jinyang Zhao, Laiyu Li, Jun Wang, Yuxin Zheng

**Affiliations:** 1 Key laboratory of Chemical Safety and Health, Chinese Centre for Disease Control and Prevention. National Institute for Occupational Health and Poison Control, Chinese Centre for Disease Control and Prevention, Beijing, 100050, China; 2 BGI-Tech, BGI-Shenzhen, Shenzhen, China; 3 Guangdong Province Hospital for Occupational Disease Prevention and Treatment, Guangzhou, China; 4 Hospital for Occupational Diseases Control of Shenzhen, Shenzhen, China; 5 Shandong Academy of Occupational Health and Occupational Medicine, Jinan, China; 6 Center for Disease Control and Prevention of Yunnan province, Kunming, Yunnan, China; 7 Food And Drug Administration Of Beijing Fengtai District, Beijing, China; 8 Health Supervision Institutionof Dongcheng Health Bureau, Beijing, China; 9 Weifang Medical University, Weifang, Shandong, China; 10 Institute of chemicals safety, Chinese academy of inspection and quarantine, Beijing, China

## Abstract

Trichloroethylene induced hypersensitivity syndrome is dose-independent and potentially life threatening disease, which has become one of the serious occupational health issues and requires intensive treatment. To discover the genetic risk factors and evaluate the performance of risk prediction model for the disease, we conducted genomewide association study and replication study with total of 174 cases and 1761 trichloroethylene-tolerant controls. Fifty seven SNPs that exceeded the threshold for genome-wide significance (*P* < 5 × 10^−8^) were screened to relate with the disease, among which two independent SNPs were identified, that is rs2857281 at *MICA* (odds ratio, 11.92; *P*_meta_ = 1.33 × 10^−37^) and rs2523557 between *HLA-B* and *MICA* (odds ratio, 7.33; *P*_meta_ = 8.79 × 10^−35^). The genetic risk score with these two SNPs explains at least 20.9% of the disease variance and up to 32.5-fold variation in inter-individual risk. Combining of two SNPs as predictors for the disease would have accuracy of 80.73%, the area under receiver operator characteristic curves (AUC) scores was 0.82 with sensitivity of 74% and specificity of 85%, which was considered to have excellent discrimination for the disease, and could be considered for translational application for screening employees before exposure.

Trichloroethylene is an industrial chemical which has been identified with neurotoxicity, hepatotoxicity, kidney toxicity, and immunotoxicity^1^,^2^,^3^. In 2012, the International Agency for Research on Cancer (IARC) classified trichloroethylene as a group I carcinogen^4^. In recent 10 years, trichloroethylene induced hypersensitivity syndrome has become one of the serious occupational health issues. The number of patients suffering from the disease has been increasing in Asia, especially the China^5^,^6^. Trichloroethylene induced hypersensitivity syndrome is dose-independent and potentially life threatening disease. The main clinical symptoms of this disease are cutaneous lesions, which ranges from mild forms such as multiform erythema to severe condition such as exfoliative dermatitis, Stevens-Johnson syndrome, and toxic epidermal necrolysis accompanying hepatitis, fever, leukocytosis and lymphadenopathy(Supplementary Fig. S1)^7^,^8^. The characteristics of this disease is quite similar to drug-induced hypersensitivity syndrome which is characterized by serious adverse systemic reaction that usually appears after 3–6 weeks of exposure to certain drugs such as anticonvulsants, dapsone and allopurinol^9^.

As we know, the rising prevalence of allergenic diseases is worldwide, hypersensitivity reactions can be induced by a diverse range of allergens in predisposed individuals. Trichloroethylene as a ubiquitous environmental contaminant in soil, water and indoor air samples and broadly-used industrial degreasing agent, extraction agent, dry cleaning agent^10^, is major pollutant that pose threat to population in both occupational and the general environment. The literature about trichloroethylene hypersensitivity syndrome published in English and especially publications in local languages were reviewed in detail by Kamijima *et al.*^5^. Occurrences of the disease have been reported from several countries including the United States, Japan, Spain, Singapore, China, Korea, Thailand, and the Philippines. Most case reports from industrialized countries were published up to 1990, whereas cases from Asian industrializing countries were published later. At present, the number of reported patients suffering from trichloroethylene hypersensitivity syndrome in China has exceeded 500, the mortality is about 9–13%^5^. So trichloroethylene hypersensitivity syndrome requires intensive treatment.

The traditional protective measures including personal protective devices, engineering controls, ventilation, work practices, and hygiene practices couldn’t reduce the incidence of trichloroethylene hypersensitivity syndrome, because that there is no obvious dose-effect relationship between the trichloroethylene exposure and incidence of the disease^11^. Therefore, screening susceptible individuals to avoid trichloroethylene exposure might be considered as priority measure to prevent the disease. Our previous results showed that trichloroethylene hypersensitivity syndrome has strong genetic linkage to human leukocyte antigen (HLA)-B*13:01 allele with odds ratio of 27.5 among exposed workers in China^12^. Further research has illustrated that HLA-B*13:01 was also identified in the patient of Japan^8^. Besides HLA-B*13:01, *tumor necrosis factor alpha* and *N-Acetyltransferase 2* were found as genetic markers for the disease^13^,^14^. However, genetic variants identified to date do not account for all cases, and there were no tests to predict the risk of trichloroethylene hypersensitivity syndrome. In this paper, we try to identify the prediction markers and evaluate the performance of risk prediction model for trichloroethylene induced hypersensitivity syndrome using data from genome-wide association study.

## Results

### Genomewide association analysis

In the discovery stage, we genotyped 2,379,855 single nucleotide polymorphism (SNP)s in 100 cases and 100 trichloroethylene-tolerant controls. After SNP-level and sample-level quality control and filtering steps, 1,392,644 SNPs in 99 cases and 99 controls were remained. Principal component analysis (PCA) showed that the cases and controls in this study were of Han Chinese ancestry and overlapped with Asian population (Supplementary Fig. S2a). There was slightly mismatch between cases and controls along principal component 1 axis after removing 2 outliers (1 case, 1 control) (Supplementary Fig. S2b and S2c), and significant difference in principal component 1 was shown by variance analysis (*P* < 0.05). Therefore, we adjusted for nominally significant eigenvector of principal component 1 using logistic regression in order to control the potential population stratification for further analysis on remained 98 cases and 98 controls. A quantile-quantile plot of the observed P values showed a clear deviation from the null distribution which likely reflect true genetic association without bias of potential population stratification (*λ*_*GC*_ = 1.013, Supplementary Fig. S3).

**Figure 1:**
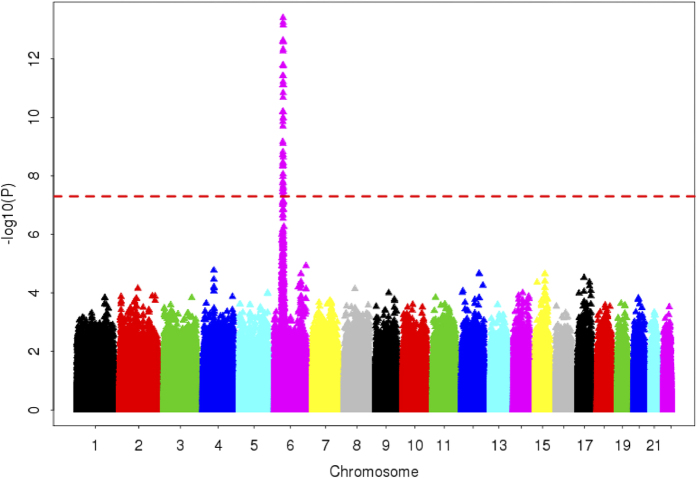
Genomewide association results. The genomewide P values of the principal component 1-adjusted logistic regression analysis from 1,392,644 SNPs in 98 cases of trichloroethylene hypersensitivity syndrome and 98 trichloroethylene-tolerant controls were presented. The chromosomal distribution of all the P values (−log_10_*P*) were shown. The red horizontal dash line represented the genomewide significance threshold of *P* = 5 × 10^−8^.

The overall association results are shown in Manhattan plot, in which we observed all the loci that exceeded the threshold for genome-wide significance (*P* < 5 × 10^−8^) are located in the *major histocompatibility complex (MHC)* region (Fig. 1). The 57 significant markers are listed in Supplementary Table S2.

### Selection of independent SNPs and tests of replication

**Figure 2:**
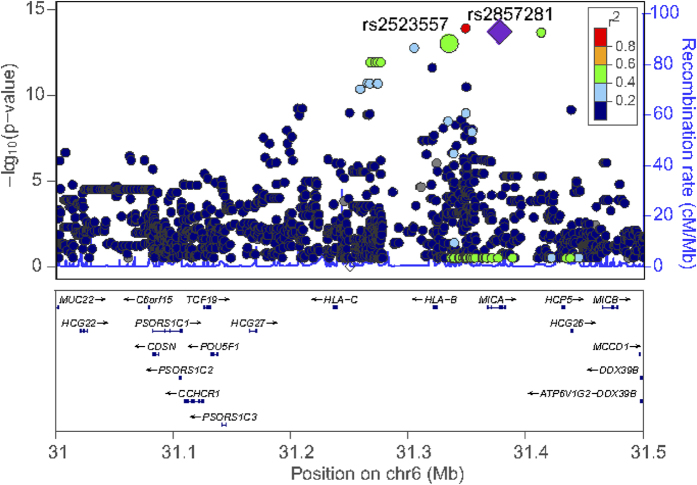
Regional association plot in the MHC for the subjects. SNPs were colored on the basis of their linkage disequilibrium (LD) with the rs2857281(highlighted with purple diamonds) which was one of the independent markers identified through conditional association study. Another identified independent marker is rs2523557 (bigger green circle). x axes, physical distance (Mb); left y axes, −log_10_*P* for association statistics. Right y axes, recombination rates, light blue line. Recombination rates were based on the 1000 Genomes Mar 2012 ASN population.

Closer examination of the significant SNPs within the MHC region showed that they are located within a 500-kb region concentrated around class I *HLA* genes (Fig. 2), but precise assignment of causal variants is challenging because of the extensive linkage disequilibrium (LD) between these SNPs. Therefore, we used conditional stepwise logistic regression to define independent markers associated with the disease. Considering of the potential biological function of each significant SNP based on their location on the chromosome and their LD with the top SNP of rs2523628, we selected rs2857281 (in *MHC class I chain related gene A (MICA)*, in strong LD with top SNP, *r*^2^ = 0.976, *D*’ = 1) instead of top SNP (intergenic between *HLA-B* and *MICA*) in step one of conditional association study. From the initial set of 57 SNPs that reached genomewide significance, two independent markers were selected for replication study, that were rs2857281 located in *MICA*(odds ratio, 24.21; *P* = 5.64 × 10^−14^) and rs2523557 located between *HLA-B* and *MICA* (odds ratio, 11.22; *P* = 2.53 × 10^−13^). (Table 1, Supplementary Table S3, Supplementary Fig. S4). These two SNPs were in moderate LD with each other (*r*^2^ = 0.468, *D*’ = 0.973) and could account for the majority of the genetic association. Rs2857281 and rs2523557 had never been previously reported to associate with hypersensitivity syndrome.

Given the localization of significant independent SNPs wholly to the *HLA* class I region, as well as previous studies showing that HLA-B*13:01 is strongly associated with the disease, we next tried to explore whether the association of the disease risk with rs2857281 and rs2523557 indirectly reflects association of HLA-B*13:01.

We then genotyped HLA-B*13:01 allele and calculated LD coefficient of *r*^2^ based on discovery data. The results showed that HLA-B*13:01 was in moderate and weak LD with rs2857281 (*r*^*2*^ = 0.74) and rs2523557 (*r*^*2*^ = 0.24), respectively, and was significantly associated with the disease risk (odds ratio, 36.97; *P*_*meta*_ = 7.77 × 10^−15^), which was stronger than the association of either rs2857281 or rs2523557. The conditional analysis revealed that association of rs2857281 and rs2523557 couldn’t cover that of HLA-B*1301, conversely, HLA-B*13:01 couldn’t cover two SNPs too (Supplementary Table S3), suggesting rs2857281 and rs2523557 were useful supplements to HLA-B*1301 for the disease risk prediction. Additional studies are needed to validate the causal association at this region.

Replication study was performed in an additional 74 cases with trichloroethylene hypersensitivity syndrome and 1661 trichloroethylene-tolerant controls. Associations of rs2857281 (odds ratio, 9.90; *P* = 5.22 × 10^−26^) and rs2523557(odds ratio, 6.41; *P* = 1.56 × 10^−23^) were successfully replicated (Table 1). In meta-analysis, rs2857281 and rs2523557 showed stronger evidence of association, with *Pmeta* of 1.33 × 10^−37^ and 8.79 × 10^−35^, respectively.

### Cumulative effects on disease risk

Cumulative effect analysis showed that the disease risk varied up to 32.5-fold for individuals with more than 2 risk alleles compared with those without risk alleles in discovery samples and 19.48-fold in replication samples (Table 2). These two SNPs cumulatively explained 52.9% of the disease variance in the discovery population and 20.9% of that in the replication population (Table 2).

### Prediction of trichloroethylene hypersensitivity syndrome risk

**Figure 3:**
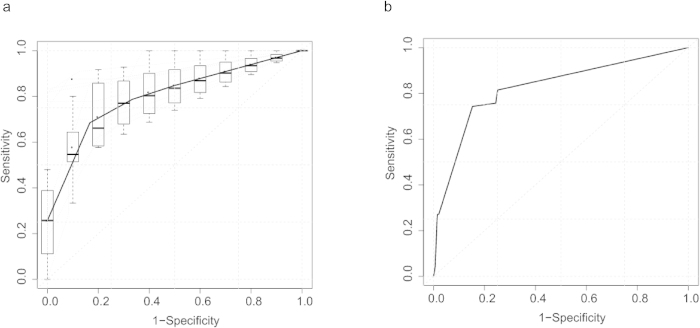
Receiver operator characteristic curves for prediction model of trichloroethylene induced hypersensitivity syndrome using two independent SNPs as predictor. **a**, Receiver operator characteristic curve of 10-fold cross validation using discovery dataset. **b**, Receiver operator characteristic curve using discovery and replication data. Discovery data was used to train prediction model, replication data was for testing data.

The genomewide association studies have been performed to detect associations between SNPs and diseases in many species. In addition to discover variants related with the disease and their biological function, disease risk predictions using results of genomewide association study are increasing concerned^15^. Therefore, we examined the potential use of rs2523557 and rs2857281 genotypes as predictive markers of the disease using logistic regression. The results showed that presence of these two SNPs had a sensitivity of 78% and specificity of 89% as risk predictors for the trichloroethylene hypersensitivity syndrome, with the AUC score of 0.85 for discovery dataset, and sensitivity of 74% and specificity of 85% with the AUC score of 0.82 for replication dataset were also found (Fig. 3, Supplementary Table S4). Generally, a test is considered predictive if the AUC is more than 0.7^16^,^17^. Combination of rs2523557 and rs2857281 as predictor would have accuracy of 80.73%, positive predictive value of 35.85% and negative predictive value of 96.96%. Taken together, the combination of rs2523557 and rs2857281 could be used in the classification of trichloroethylene exposed populations with risk of developing hypersensitivity syndrome.

## Discussion

Trichloroethylene induced hypersensitivity syndrome is considered to be T-cell mediated immune diseases, in which potential genetic factors including HLA background, immune cytokine and chemokines polymorphisms, as well as polymorphisms of cell surface receptors are involved. In present study, we not only validate the association between HLA-B*13:01 allele and trichloroethylene hypersensitivity syndrome, but also identified two new loci for the disease, one is on intron of *MICA*, and another is between *HLA-B* and *MICA*, suggesting *MICA* is an important gene for the disease risk in addition to HLA-B*13:01.

*MICA,* the major histocompatibility complex class I chain - related gene A, is characterized by high degree of polymorphism, which encoded protein are involved in immune surveillance^18^,^19^. Although the association of *MICA* with autoimmune and neoplastic diseases, including ankylosing spondylitis^20^, Behcet’s disease^21^, psoriasis vulgaris^22^, and Kawasaki’s disease^23^, have been defined, there are no reports for the association with hypersensitivity dermatitis. *MICA* is tightly regulated and is expressed constitutively in intestinal epithelial cells under normal conditions. However, through a poorly defined mechanism, MICA expression is up-regulated on cells stressed by infection or malignant transformation in many tissues^24^. MICA may stimulate a cellular immune response by interacting with cell surface receptor natural killer group 2 membrane D (NKG2D), which is expressed on natural killer cells (NK), natural killer T cells (NKT), CD8^+^T cells, and γδT cells. The tissue cells expressing MICA are recognized and attacked by NKG2D bearing immune cells and induce tissue pathological injury^25^. Kamijima M *et al.* reported that human herpesvirus 6 reactivation was identified in 89% patients with trichloroethylene hypersensitivitydermatitis^26^. Human herpesvirus 6 infects most children between 6 months and 2 years of age to cause exanthema subitum, and then remains latent after primary infection. This result supposed that virus reactivation after trichloroethylene exposure might be a stress on the cells and induced the expression of MICA, which in turn to participate in the development of the disease through MICA-NKG2D signal pathway. The association of *MICA* offers new insights into the mechanisms of genetic susceptibility to trichloroethylene hypersensitivity syndrome, while further studies are needed to investigate its functional relevance to the disease.

To our knowledge, this is the first genomewide association study about hypersensitivity syndrome induced by industrial chemicals, and two novel SNPs were found to strongly associate with the disease, the highest odds ratio was 11.92 for meta-analysis, which is rare in this kind of studies. Although genomewide association studies were broadly performed to identify susceptibility loci for many complex diseases such as cancer^27^, diabetes^28^,^29^,^30^, cardiovascular disease^31^, allergic diseases^32^, etc, and provided valuable insights into their genetic components, however, most of the studies have explained little of heritability, and the associated variants have small effect sizes^33^. The missing heritability is possibly caused by unmapped common and rare variants, copy number of variations, epigenetic effects, gene-gene interactions and gene-environment interactions^34^. It suggests that interaction between genetic and environmental factors may play an important role in the aetiology of the most diseases, however, the degree to which genetic and environmental factors influence susceptibility to the diseases is not well defined. One main feature of occupational diseases is relatively defined causative factors, so studies on this kind of diseases are helpful in identifying the role of genetic factors and providing the available biomarkers for screening employees before exposure. Therefore, selection of occupational population with defined exposure for identifying genetic susceptibility is the advantage of our study, and the identified two SNPs could explain 20.9% of the disease variance. The combination of these two SNPs could be effective predicting biomarker for hypersensitivity syndrome among trichloroethylene exposed population and could be considered for translational application for susceptible individual screening.

We fully recognize that our study has some limitations. First, there was no data about the functions of the identified SNPs, especially the rs2857281, which located on intron of MICA. Second, small number of cases was involved in this study, although this is the largest available sample of trichloroethylene hypersensitivity syndrome patients in the world with accurate history of trichloroethylene exposure. The study power calculation revealed that the study was powerful enough to detect the variants with such strong effects, namely the power exceeded 93% to detect variants with an allele frequency above 5% and odds ratio above 10.0 at genome-wide significance of 5 × 10^−8^ (Supplementary Table S5).

The risk allele frequencies of C at rs2857281 and C at rs2523557 for trichloroethylene-tolerant controls in southern Chinese of present study (8.1% and 13.0%, respectively) are similar to that of east Asian (8.04% and 15.03%, respectively, 1000 Genomes ASN population, n = 286). The higher frequencies of risk allele for both SNPs were observed in African (17.89% and 34.35%, respectively, 1000 Genomes AFR, n = 246) and European (7.52% and 33.61%, respectively, 1000 Genomes EUR, n = 379)^35^. Given the higher risk allele frequencies for majority of the population and broadly usage of trichloroethylene in the world wide, trichloroethylene induced hypersensitivity syndrome should be given more attention all over the world.

In conclusion, we provide evidence that two new loci within the HLA region in addition to HLA-B*13:01 are significantly associated with trichloroethylene hypersensitivity syndrome, and could explain approximately 20.9% of the disease variance. The combination of these SNPs could be an effective predicting biomarker for the disease among trichloroethylene-exposed populations. Further work is needed to study the biological meaning of the association between the newly described SNPs and the disease.

## Methods

### Ethics statement

All experiments in this study were approved by the Research Ethic Committee of Guangdong Province Hospital for Occupational Disease Prevention and Treatment, and all participants signed an informed consent. The methods were carried out in accordance with the approved guidelines.

### Study subjects and sample collection

We performed two-stage genomewide association study. The subjects for discovery (100 cases with trichloroethylene hypersensitivity syndrome and 100 trichloroethylene-tolerant controls) and for replication (74 cases and 1661 tolerant controls) were recruited in 1998s–2006s and 2007s–2011s, respectively. All of them were unrelated individuals from 351 factories engaged in electronic-element and metal–plating production in Guangdong Province, China. Both cases and controls were exposed to trichloroethylene in the workplace where they cleaned and degreased metals. All the cases diagnosed by the panel of occupational physicians according to Chinese National Diagnostic Criteria of Occupational Disease. The latency of trichloroethylene hypersensitivity syndrome ranges from 4 to 77 days. Controls were defined as the co-workers of the patients with same job title and longer occupational exposure time (>3 months) but no skin abnormalities detected by the occupational physicians upon examination. The epidemiologic data showed that if worker does not suffer from trichloroethylene hypersensitivity syndrome within 3 months from beginning of trichloroethylene exposure, the worker is trichloroethylene-tolerant^36^. Therefore, all controls in present study were confirmed as trichloroethylene-tolerant workers without predisposition of the disease.

All participants signed an informed consent form before commencement of the study. The demographic information for subjects were collected using the questionnaire and ancestry was determined by self-reported. After informed consent was obtained, peripheral blood samples were collected for DNA extraction. The age, sex, exposure duration for subjects is summarized in Supplementary Table S1.

### Genotyping of single nucleotide polymorphisms and association analysis.

We performed the genomewide association study using HumanOmin2.5-8 BeadChip (Illumina) and the follow-up genotyping using Sanger sequence analysis on ABI3730xl capillary sequencing machines (Applied Biosystems). HLA-B*13:01 genotyping was detected by primer specific polymerase chain reaction- restriction fragment length polymorphism method. Single nucleotide polymorphism (SNP) quality control and sample quality control were done by R package, and population stratification was assessed by principal component analysis.

Association analyses were performed using log additive logistic regression model in PLINK software^37^ (version 1.07) with correction for population stratification because of the slightly mismatch between cases and controls. We selected independent SNPs from the SNPs that reached genomewide significance in discovery stage using conditional stepwise logistic regression. The meta-analysis was performed using the fixed-effects model (Mantel-Haenszel model). Heterogeneity was examined using the χ^2^-based Cochran’s Q statistic. The genetic risk score was calculated as a weighted sum of the number of risk alleles at each locus multiplied by the log of the odds ratio for each of the individual loci, and the percentage of the total variance explained by the genetic risk score was estimated by Nagelkerke’s pseudo γ^2^. Disease risk prediction model was built using logistic regression according to the method described by Zhi Wei *et al.*^38^ and evaluated the performance of the model by the area under receiver operator characteristic curves (AUC) scores from 10-fold cross-validation and independent dataset validation. More information about the subjects, genotyping, quality control, statistical analyses is provided in the Supplementary Appendix.

## Additional Information

**How to cite this article**: Dai, Y. *et al.* Performance of genetic risk factors in prediction of trichloroethylene induced hypersensitivity syndrome. *Sci. Rep.* **5**, 12169; doi: 10.1038/srep12169 (2015).

## Supplementary Material

Supplementary Information

## Figures and Tables

**Table 1:** Association results for the independent SNPs in the MHC region identified by conditional association study.

SNP	Chr	Location & Related Genes	Allele (A1/A2)	Study	Frequency in cases	Frequency in tolerant controls	Odds Ratio_add_^a^ (95%CI)	P add^a^	P_het_^b^
rs2857281	6	intronic, MICA	C/A	Discovery (98cases, 98controls)	0.41	0.056	24.21 (10.55,55.59)	5.64 × 10^−14^	0.061
				Replication (71cases, 1661controls)	0.44	0.082	9.90 (6.47,15.15)	5.22 × 10^−26^	
				Meta-analysis (169cases, 1759controls)	0.42	0.081	11.92 (8.16,17.42)	1.33 × 10^−37^	
rs2523557	6	intergenic, HLA-B(dist = 6268), MICA (dist = 40114)	C/T	Discovery (98cases, 98controls)	0.59	0.107	11.22 (5.871,21.44)	2.53 × 10^−13^	0.140
				Replication (72cases, 1659controls)	0.50	0.131	6.41 (4.45,9.23)	1.56 × 10^−23^	
				Meta-analysis (170cases, 1757controls)	0.55	0.130	7.33 (5.34,10.07)	8.79 × 10^−35^	
HLA-B*13:01	6	HLA-B	+/−	Discovery (98cases, 98controls)	0.72	0.102	36.97 (14.87,91.89)	7.77 × 10^−15^	0.742
				Replication (69cases, 1589controls)	0.83	0.096	44.58 (23.40,84.92)	7.46 × 10^−31^	
				Meta-analysis (167cases, 1687controls)	0.76	0.097	41.88 (24.75,70.87)	5.02 × 10^−44^	

^a^Odds Ratio_add_ and *Padd* values were computed for principal component 1-adjusted logistic regression models. ^b^Heterogeneity across studies was examined using the Cochran’s Q statistics. The odds ratio and frequency is given for the A1 allele, where odds ratio >1 indicates an adverse effect. Meta-analysis was performed using the fixed-effects model. Chr., Chromosome; C, cytosine; G, guanine; T, thymine; A, adenine. CI, Confidence interval.

**Table 2:** Cumulative effect of two independent loci stratified by the number of risk alleles.

Number of risk alleles	Discovery (n = 196)* 98 cases / 98 controls			replication (n = 1729)* 70 cases / 1659 controls		
	Number (cases/controls)	Genetic risk score (mean ± SD)	Odds Ratio (95%CI)	Number (cases/controls)	Genetic risk score (mean ± SD)	Odds Ratio (95%CI)
0 (lowest risk)	18/78	0.000	1(reference)	13/1242	0.00	1(reference)
1	5/10	2.42 ± 0.00	2.17 (0.66,7.12)	5/162	1.90 ± 0.13	2.95 (1.04,8.38)
2+ (highest risk)	75/10	6.89 ± 1.86	32.50 (14.09,74.94)	52/255	4.52 ± 0.99	19.48 (10.45,36.31)
Odds Ratiochange^a^			32.50			19.48
P value, Odds Ratio (95%CI)^b^			9.11 × 10^−15^, 4.37 (3.01,6.34)			<2.0 × 10^−16^, 3.42 (2.72,4.29)
Nagelkerke^c^* r*^2^			52.9%			20.9%

*Risk scores were calculated on the basis of the Odds Ratio and allele frequencies for each stage study. Only individuals with nonmissing genotypes for all two alleles were included in this analysis. ^a^Fold-change in Odds Ratio between highest and lowest risk group. ^b^*P* value and Odds Ratio for the genetic risk score prediction model. ^c^Nagelkerke’s pseudo *r*^2^ indicates fraction of the variance in risk explained by the risk score model. SD, standard deviation; CI, Confidence interval.
