# Supplementary material for: Performance of genetic risk factors in prediction of trichloroethylene induced hypersensitivity syndrome
Source: Sci Rep. 2015 Jul 20;5:12169. doi: 10.1038/srep12169 (PMC4507183; doi:10.1038/srep12169)
Supplement: Supplementary Information [file srep12169-s1.doc]

**Supplementary Information**

**Performance of genetic risk factors in prediction of trichloroethylene induced hypersensitivity syndrome**

Yufei Dai^1^, *, Ying Chen^2^, *, Hanlin Huang^3^, *, Wei Zhou^4^, Yong Niu^1^, Mingrong Zhang ^2^, Ping Bin^1^, Haiyan Dong^6^, Qiang Jia^5^, Jianxun Huang^3^, Juan Yi^4^, Qijun Liao^2^, Haishan Li^10^, Yanxia Teng^8^, Dan Zang^7^, Qingfeng Zhai^9^, Huawei Duan^1^, Juan Shen^2^, Jiaxi He^4^, Tao Meng^1^, Yan Sha^4^, Meili Shen^1^, Meng Ye^1^, Xiaowei Jia^1^, Yingping Xiang^4^, Huiping Huang^4^, Qifeng Wu^3^, Mingming Shi^2^, Xianqing Huang^4^, Huanming Yang ^2^, Longhai Luo^2^, Sai Li^2^, Lin Li^2^, Jinyang Zhao^2^, Laiyu Li^3, #^, Jun Wang ^2, #^, Yuxin Zheng^1, #^

^1^ Key laboratory of Chemical Safety and Health, Chinese Centre for Disease Control and Prevention. National Institute for Occupational Health and Poison Control, Chinese Centre for Disease Control and Prevention, Beijing, 100050, China.

^2^ BGI-Tech, BGI-Shenzhen, Shenzhen, China.

^3^ Guangdong Province Hospital for Occupational Disease Prevention and Treatment, Guangzhou, China.

^4^ Hospital for Occupational Diseases Control of Shenzhen, Shenzhen, China.

^5^ Shandong Academy of Occupational Health and Occupational Medicine, Jinan, China.

^6^ Center for Disease Control and Prevention of Yunnan province，Kunming, Yunnan, China.

^7^ Food And Drug Administration Of Beijing Fengtai District，Beijing, China.

^8^ Health Supervision Institution of Dongcheng Health Bureau, Beijing, China.

^9^ Weifang Medical University, Weifang, Shandong, China.

^10^ Institute of chemicals safety，Chinese academy of inspection and quarantine, Beijing ,China.

**Correspondence to:**

Yuxin Zheng PhD

Key Laboratory, National Institute for Occupational Health and Poison Control

Chinese Center for Disease Control and Prevention

Nanwei Road 29, Beijing, P.R. China, 100050

Telephone: 8610-83132515

Fax: 8610-83132681

E-mail: yx_zheng@139.com

Or to Laiyu Li, Professor, M.D.

Institute of toxicology, Guangdong Province Hospital for Occupational Disease Prevention and Treatment

68 Haikang St., Xingang Rd. W., Haizhu District, Guangzhou, P. R. China, 510300

Telephone: (86)13662533086

E-mail: 13662533086@139.com

Or to Jun Wang,

BGI-Tech, BGI-Shenzhen

Yantian District, 518083 Shenzhen, China.

E-mail: wangj@genomics.cn

^*^The authors contributed equally to this work

**Supplementary Methods**

**Study subjects.** We performed a two-stage GWAS. The subjects for GWAS (100 cases with trichloroethylene hypersensitivity syndrome and 100 trichloroethylene-tolerant controls) and for replication (74 cases and 1661 controls) were recruited in 1998s-2006s and 2007s-2011s, respectively. All samples from both the discovery and replication stages were unrelated individuals of Chinese descent. All of them were from 351 factories engaged in electronic-element and metal–plating production in Guangdong Province, China.

Both cases and controls were exposed to trichloroethylene in the workplace where they cleaned and degreased metals. All the cases recruited by the diagnostic criteria proposed by a panel of occupational physicians according to Chinese National Diagnostic Criteria of Occupational Disease: (1) showed a rash on the extremities, face, neck or trunk with or without fever; (2) symptoms appearing between 4-77 days after occupational trichloroethylene exposure commenced; (3) eliminated other diseases or drugs induced similar symptoms. Controls were defined as the co-workers of the patients with same job title and longer occupational exposure time (> 90 days) but no skin abnormalities detected by the occupational physicians upon examination.

The clinical and demographic information for all subjects were collected using the questionnaire and ancestry was determined by self-reported. Ancestries of the samples used in the discovery stage were further confirmed through PCA analysis (see below and Supplementary Fig. S2). The age, sex, exposure duration for all subjects on GWAS and replication are summarized in Supplementary Table S1.

**Genomewide genotyping and genotype quality control.** DNA was extracted from whole blood samples using standard methods. Discovery samples were genotyped using the Illumina HumanOmin2.5-8 beadchip (2,379,885 markers) according to the manufacturer’s instructions at BGI-shenzhen, China. Genotyping calling was done with normalized intensities using the Illumina clustering algorithm (GenTrain 1.0) implemented in GenomeStudio Genotyping Analysis Module (v1.6.3). Approximately equal number of cases and controls were genotyped in each batch to protect against potential technical artifacts leading to differential bias in the analysis. SNP quality control and sample quality control were done by GENEVA R package (<http://www.genevastudy.org/>).

All individuals were assessed by checking their call rate, gender, and potential relatedness among samples (using pairwise identity-by-descent coefficients using Maximum Likelihood Estimation implemented in GENEVA R package). Samples with potential 1st and 2st degree family relationship were assessed for their call rates and the ones with lower call rate were removed (2 cases and 2 controls were duplicates or monozygotic twins, two of them were removed). There were no samples with call rate less than 0.95. After these quality control filters, there were 99 cases and 99 controls remained. Sex of each sample was determined by combining analysis of allelic probe intensities and called genotypes. The imputed gender was compared to self-reported gender, and two mismatched samples were corrected.

Ancestry and population stratification were assessed using principal component analysis (PCA) on the remained 198 samples. We randomly selected a set of 10,000 SNPs within which each pair has a low level of linkage disequilibrium, from a starting pool of autosomal SNPs with missing call rate < 0.05. The first round of principal component analysis was carried out on all 198 samples and 618 individuals from four populations (CHB, JPT, CEU, YRI) from HapMap3 project (see URLs) to evaluate the population structure (Supplementary Fig. S2a). The second round of principal component analysis was to identify population outlers, and then third round of principal component analysis was utilized to assess the genetic mismatch between cases and controls after removing the outliers. We plot the first three eigenvectors in a pairwise fashion for each individual by coloring with disease status (case in red and control in blue) (Supplementary Fig. S2). Meanwhile, variance analysis was used to test principal components difference between cases and controls for first three principal components. We included principal component 1 as covariant in logistic regression to control population stratification in all next analysis, because that principal component 1 showed a nominally significant association with case-control status (P<0.05, Supplementary Table S2). To investigate potential residual population stratification, we calculated genomic control inflation factors and inspected quantile-quantile plots of test statistics for both all SNPs and the SNPs outside the MHC region.

SNP quality control filters were applied as following criterions: (1) removal of non-autosomal and intensity-only SNPs; (2) removal SNPs with call rate < 95%; (3) minor allele frequency < 0.25% in cases and controls; (4) significant from deviation from Hardy-Weinberg equilibrium in the controls (*P* < 10^-4^). A total of 1,392,664 (58.52%) SNPs were finally remained for genome-wide association study with a mean call rate of 0.9982. Cluster plots of all significant SNPs considered for replication (*P* ≤ 5×10^-8^) were also manually inspected for quality assurance.

In summary, after quality control and filtering processes, genotype data for 1,392,664 SNPs in 98 cases and 98 controls were used for the genomewide association analysis.

**SNP selection for replication stages.** To select SNPs for the replication analysis, we first verified top 57 SNPs that reached our *P*-value threshold by visual inspection of the Illumina cluster plots manually, and then we carried out stepwise logistic regression by controlling for the genotypes of the conditioning SNPs using PLINK (v 1.07) to detect independent signals. In total, we selected 2 representative SNPs for genotyping in replication study of 74 cases and 1661 controls. The association analysis of the 2 SNPs (rs2857281, rs2523557) and *HLA-B*13:01* was performed using logistic regression analysis in replication samples.

***HLA-B*13:01* genotyping.** In replication stage, *HLA-B*13:01* allele was detected by polymerase chain reaction- restriction fragment length polymorphism method. In brief, first the entire gene was amplified with *HLA-B* specific primers of 5’-TGGGCTACGTGGACGACA-3’ and 5’ **-**TCCTGATCTGAGTGGAGGTAAAG-3’ after which the ‘long’ PCR product was used as a template in consequent nested–PCRs using *HLA-B*13* specific primers of 5’-TACCGAGAGAACCTGCGCA-3’ and 5’-GGCCGCCTCCCACTTGA-3’. The polymerase chain reaction was performed using Premix Taq® Version 2.0 (Takara Bio Inc, Japan). The carriers with 476bp nested polymerase chain reaction product are *HLA-B*13* positive subjects. In order to distinguish *HLA-B*13:01*, 10 ul of nested polymerase chain reaction products were digested by FokI restriction enzyme at 37℃ for 10 h and electrophoresis was performed in 2% agarose gel. The 476 bp PCR product is cut into 77bp, 217bp, 35bp, and 147bp among *HLA-B*13:01* positive subjects.

The successful genotyping datasets consist of 1658 samples with 69 cases and 1589 controls for *HLA-B*13:01* allele.

**Genotyping of rs2523557 and rs2857281 on replication study.** Genotypes for 2 SNPs, rs2523557 and rs2857281, selected for follow-up analysis were conducted at Core Genomic Facility, Beijing Institute of Genomics, Chinese Academy of Sciences. The sample DNA from 74 cases and 1661 controls was amplified by PCR reaction and the PCR products were then subjected to Sanger sequence analysis on ABI3730xl capillary sequencing machines (Applied Biosystems, Foster City, CA, USA). The successful genotyping datasets consist of 1732 samples with 71 cases and 1661 controls for rs2857281, 1731 samples with 72 cases and 1659 controls for rs2523557, respectively.

**Statistical analysis.** The association analysis between SNPs (or *HLA* allele) and trichloroethylene hypersensitivity syndrome was carried out using logistic regression under a log additive model in PLINK v1.07 (http://pngu.mgh.harvard.edu/~purcell/plink/). To test the additive model for each SNP, we encoded 0, 1 and 2 for homozygous for major allele, heterozygous and homozygous for minor allele, respectively. For *HLA-B**13:01, we labeled *HLA-B**13:01-positive for individuals with 1 or 2 *HLA-B**13:01 allele and *HLA-B**13:01-negative for those without *HLA-B**13:01 allele. In discovery stage, the principal component 1 was included as covariate to adjust for population stratification. Odds ratio per copy risk allele, 95% confidence intervals (CIs), and corresponding *P*-values were presented. A *P-*value cutoff of 5×10^-8^ was used to declare statistical significance. The quantile-quantile plot and Manhattan plot were generated using the R software. The regional plot was plotted using an online tool, LocusZoom 1.1 (see Uniform Resource Location). Heterogeneity across studies was examined using the χ^2^-based Cochran’s Q statistic. Meta-analysis was performed using the fixed-effects model (Mantel-Haenszel model) if the result of the heterogeneity test showed P_het_ ≥ 0.05 or using the random-effects model (DerSimonian and Laird model) if P_het_ < 0.05.

**Genetic risk score and variance explained.** The genetic risk score was calculated on risk alleles for the two independent and most strongly associated SNPs (rs2857281, rs2523557). The genetic risk score was calculated as a weighted sum of the number of risk alleles at each locus multiplied by the log of the odds ratio for each of the individual loci for discovery and replication stage. Only individuals with non-missing genotypes for all two alleles were included in this analysis.

The percentage of the total variance explained by the genetic risk score was estimated by Nagelkerke’s pseudo *r*^2^ from the logistic regression model with the genetic risk score as a quantitative predictor and disease status as an outcome.

**Rs2857281 and rs2523557 as risk predictors of trichloroethylene hypersensitivity syndrome**

**Risk prediction model building.** Risk prediction model was built according to the method described by Zhi Wei et al[^1^](#_ENREF_1). In this study, genotypes on *p* SNPs for *n* individuals could be presented by an *n* * *p* matrix G = (*g_ij_*), where *g_ij_* indicates the genotype of SNP *i* for individual *j*. The logistic regression was used to build disease risk prediction model, which modeled the posterior probabilities of being a case or a control via a linear combination of SNP genotypes. Under the logistic regression model, the probability of being cases (*y* =1) is exp (â^T^g) / (1+exp (Error: Reference source not found)), where â = (*â_0_*, *â_1_*, … ,*â_p_*) and g=(*1, g_1_*, … , *g_p_*)^T^. Given the training data, the logistic regression model is fit to get a maximum likelihood estimate (MLE) of â, and this estimate can be used for future prediction.

**Evaluation of risk assessment model.** The performance of the risk prediction model was evaluated by the area under receiver operator characteristic curves (AUC) scores from k-fold cross-validation and independent validation[^1^](#_ENREF_1). The k-fold cross-validation is most widely used method for estimating prediction performance. In k-fold cross-validation, the data are divided into k equal parts, and the model is fit on k-1/k of the data (training data) and then tested on 1/k of the remaining data (testing set). This is repeated for each possible k-1/k and 1/k of the data, and predictions are obtained. In this study, we do 10-fold cross-validation based on discovery data (within-study cross-validation) to evaluate risk prediction model. The independent replication dataset was subsequently evaluated in order to assess the prediction model in an unbiased way. In the independent validation of risk prediction model, the discovery data is used for training data and the replication data is for testing data.

Receiver operator characteristic curves display the relationship between sensitivity (true positive rate) and 1-specitivity (false positive rate) across all possible threshold values that defined the positivity of a condition. AUC indicated the discriminative accuracy for persons who will develop the disease or not. AUC ranges from 0.5 to 1.0, the higher the better. In general, AUC of greater than 0.8 was considered to have excellent discrimination and 0.7 acceptable discrimination. The receiver operator characteristic curve and AUC was performed by ROCR package (http://cran.r-project.org/web/packages/ROCR/index.html).

**Power analysis.** The power analysis was carried out using methods described[^6^](#_ENREF_6). The calculations were carried out under the following assumption: a disease prevalence of 1%; an additive risk model with sample size of 100 cases and 100 controls, and significance level of 5×10^-8^. The power of our study design was calculated for a range of risk allele frequencies (0.04-0.10) and effect sizes (odds ratio 1.20-10.0). The power calculations and the needed number of cases at α = 5 × 10^-8^ and a power of 0.80 were estimated using GPC software (http://pngu.mgh.harvard.edu/~purcell/gpc/) (Supplementary Table S5). Power analysis showed that we have good probability of finding a variant with effect ≥ 10 and allele frequency ≥ 0.04 or effect > 5 and allele frequency ≥ 0.15.

Supplementary References

1. Wei, Z. *et al.* From disease association to risk assessment: an optimistic view from genome-wide association studies on type 1 diabetes. *PLoS Genet* **5**, e1000678 (2009).

2. Jakobsdottir, J., Gorin, M.B., Conley, Y.P., Ferrell, R.E. & Weeks, D.E. Interpretation of genetic association studies: markers with replicated highly significant odds ratios may be poor classifiers. *PLoS Genet* **5**, e1000337 (2009).

3. Kraft, P. *et al.* Beyond odds ratios--communicating disease risk based on genetic profiles. *Nat Rev Genet* **10**, 264-9 (2009).

4. Aittokallio, J. *et al.* Overnight variability in transcutaneous carbon dioxide predicts vascular impairment in women. *Exp Physiol* **93**, 880-91 (2008).

5. Janssens, A.C. & van Duijn, C.M. Genome-based prediction of common diseases: methodological considerations for future research. *Genome Med* **1**, 20 (2009).

6. Skol, A.D., Scott, L.J., Abecasis, G.R. & Boehnke, M. Joint analysis is more efficient than replication-based analysis for two-stage genome-wide association studies. *Nat Genet* **38**, 209-13 (2006).

**Supplementary figure**


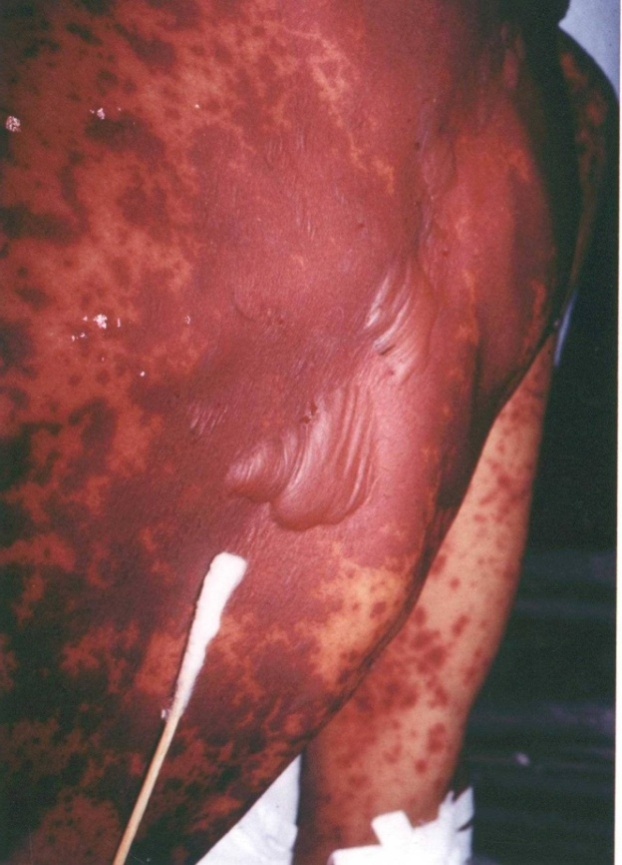

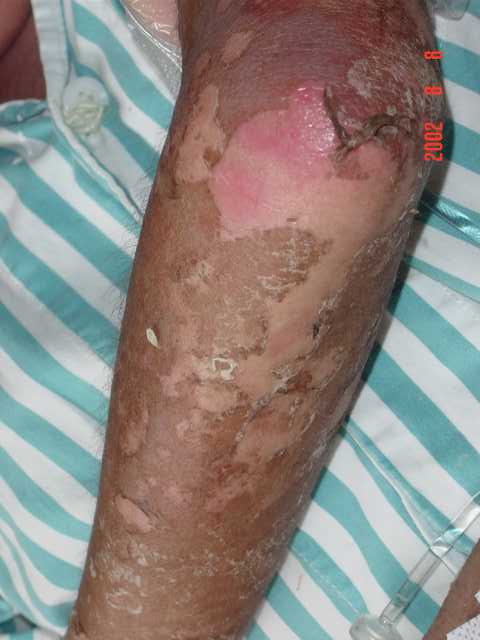

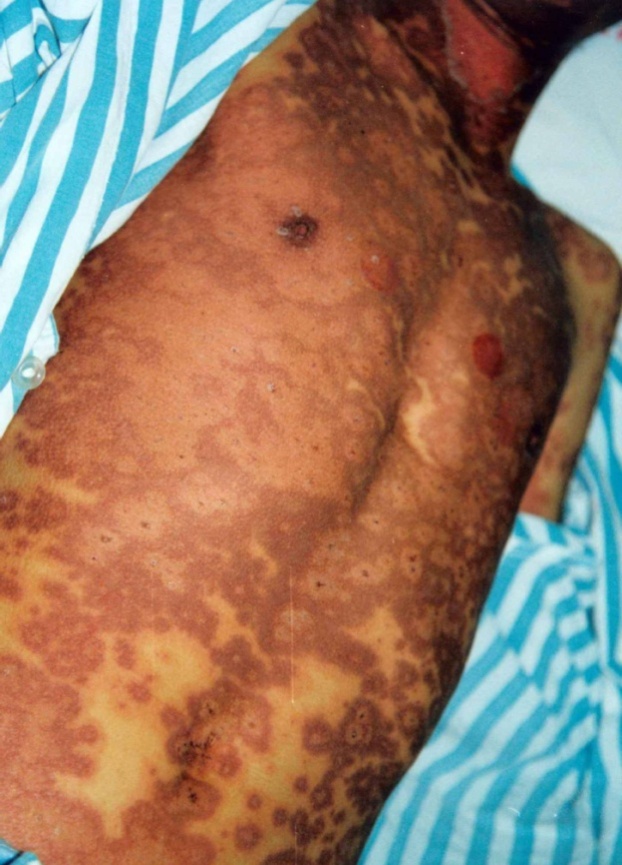


**A**

**B**

**C**

**Figure S1. Performance of skin lesions in patients with trichloroethylene hypersensitivity syndrome.** A. toxic epidermal necrolysis. B. exfoliative dermatitis. C. multiform erythema.

a PC1 vs PC2 (198 GWAS samples and HapMap3 618 individuals (CEU, CHB+JPT, YRI)


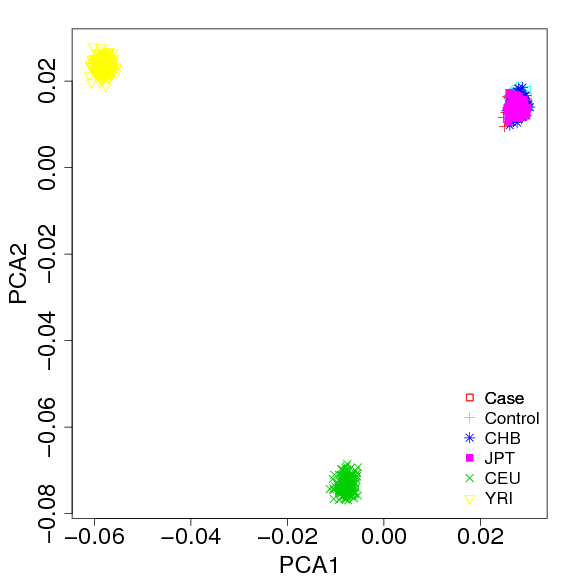


b1 PC1 vs PC2 (198 samples)

c1 PC1 vs PC3 (198 samples)

b2 PC1 vs PC2 (196 samples)

c2 PC1 vs PC3 (196 samples)

**Figure S2. Plots of first three principal components**. (a) The principal component analysis (PCA) of GWAS samples and HapMap3 618 individuals (165 CEU, 250 CHB+JPT, 203 YRI). (b1) and (c1) Plot of first three principal components for 198 samples (99 cases and 99 controls) after SNP-level and sample-level quality control. (b2) and (c2). Plot of first three principal components for 196 samples (98 cases and 98 controls) after removing 2 outliers.


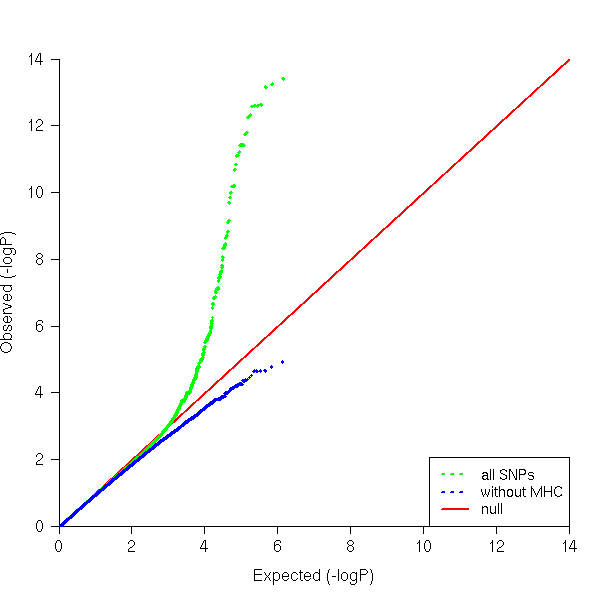


**Figure S3. Quantile-qauntile plot of the association results with (green) and without (blue) SNPs in MHC.**

**Figure S4. Cluster plots of genotype calls for two independent SNPs.**

**Supplementary table**

### Table S1. Sample summary information in the genomewide association studies

| Variable | Discovery | | Replication | | Meta-analysis | |
| --- | --- | --- | --- | --- | --- | --- |
|  | cases | controls | cases | controls | cases | controls |
| Sample size | 100 | 100 | 74 | 1661 | 174 | 1761 |
| Age[years, (mean±SD)] | 24.07±7.00 | 24.00±5.56 | 22.85±5.67 | 27.121±8.31 | 23.55±6.48 | 26.94±8.21 |
| Male/female(%) | 54.0/46.0 | 48.0/52.0 | 56.8/43.2 | 57.6/42.4 | 55.7/44.3 | 57.0/43.0 |
| TCE exposure duration (days (min, max) ) | 28(4-70) | >90 | 30(5-77) | >90 | 29(4-77) | >90 |

### Table S2. Association results for 57 SNPs that reach genome-wide significance (*P*< 5×10^-8^)^#^

| CHR | SNP | BP | Min/Maj | MAF case/control | Odds Ratio[95%CI] | *P* | Location&RelatedGenes |
| --- | --- | --- | --- | --- | --- | --- | --- |
| 6 | rs2523628 | 31346359 | T/C | 0.42/0.06 | 23.63[10.41,53.64] | 3.94×10^-14^ | intergenic,HLA-B(dist=21370),MICA(dist=25012) |
| 6 | rs2857281 | 31374262 | C/A | 0.41/0.06 | 24.21[10.55,55.59] | 5.64×10^-14^ | intronic,MICA |
| 6 | rs2844510 | 31410408 | A/C | 0.42/0.06 | 23.92[10.42,54.92] | 7.00×10^-14^ | intergenic,MICA(dist=27318),HCP5(dist=20549) |
| 6 | kgp1819618 | 31320849 | C/T | 0.43/0.09 | 14.70[7.163,30.17] | 2.33×10^-13^ | downstream,HLA-B |
| 6 | rs2523559 | 31330969 | G/T | 0.59/0.11 | 11.22[5.87,21.44] | 2.53×10^-13^ | intergenic,HLA-B(dist=5980),MICA(dist=40402) |
| 6 | rs2523557 | 31331257 | C/T | 0.59/0.11 | 11.22[5.87,21.44] | 2.53×10^-13^ | intergenic,HLA-B(dist=6268),MICA(dist=40114) |
| 6 | kgp8779347 | 31409802 | T/A | 0.39/0.06 | 23.37[10.04,54.39] | 2.66×10^-13^ | intergenic,MICA(dist=26712),HCP5(dist=21155) |
| 6 | kgp5542250 | 31412447 | G/A | 0.38/0.06 | 22.45[9.66,52.17] | 4.75×10^-13^ | intergenic,MICA(dist=29357),HCP5(dist=18510) |
| 6 | rs9500887 | 31302591 | G/T | 0.38/0.06 | 20.23[8.94,45.81] | 5.48×10^-13^ | intergenic,HLA-C(dist=62678),HLA-B(dist=19058) |
| 6 | kgp11163133 | 31352086 | C/T | 0.47/0.13 | 11.25[5.75,22.03] | 1.61×10^-12^ | intergenic,HLA-B(dist=27097),MICA(dist=19285) |
| 6 | kgp17159519 | 31435428 | G/A | 0.37/0.07 | 17.14[7.78,37.76] | 1.76×10^-12^ | intergenic,HCP5(dist=1842),HCG26(dist=3578) |
| 6 | rs9461685 | 31265355 | C/T | 0.36/0.06 | 16.78[7.57,37.19] | 3.82×10^-12^ | intergenic,HLA-C(dist=25442),HLA-B(dist=56294) |
| 6 | rs9295970 | 31269522 | T/A | 0.36/0.06 | 16.78[7.57,37.19] | 3.82×10^-12^ | intergenic,HLA-C(dist=29609),HLA-B(dist=52127) |
| 6 | rs10081114 | 31271469 | C/T | 0.36/0.06 | 16.78[7.57,37.19] | 3.82×10^-12^ | intergenic,HLA-C(dist=31556),HLA-B(dist=50180) |
| 6 | rs9468942 | 31274441 | A/G | 0.36/0.06 | 16.78[7.57,37.19] | 3.82×10^-12^ | intergenic,HLA-C(dist=34528),HLA-B(dist=47208) |
| 6 | kgp11347311 | 31351572 | G/T | 0.55/0.11 | 8.88[4.77,16.55] | 6.11×10^-12^ | intergenic,HLA-B(dist=26583),MICA(dist=19799) |
| 6 | kgp8529577 | 31407201 | T/G | 0.44/0.12 | 10.49[5.35,20.56] | 7.76×10^-12^ | intergenic,MICA(dist=24111),HCP5(dist=23756) |
| 6 | kgp9593289 | 31407257 | A/G | 0.44/0.12 | 10.49[5.35,20.56] | 7.76×10^-12^ | intergenic,MICA(dist=24167),HCP5(dist=23700) |
| 6 | rs2844586 | 31318024 | T/C | 0.38/0.09 | 12.14[5.94,24.82] | 7.83×10^-12^ | intergenic,HLA-C(dist=78111),HLA-B(dist=3625) |
| 6 | kgp9149991 | 31407218 | A/G | 0.44/0.12 | 9.63[4.99,18.58] | 1.45×10^-11^ | intergenic,MICA(dist=24128),HCP5(dist=23739) |
| 6 | rs4143334 | 31348200 | C/T | 0.44/0.14 | 9.67[4.98,18.78] | 2.10×10^-11^ | intergenic,HLA-B(dist=23211),MICA(dist=23171) |
| 6 | rs9468929 | 31263216 | A/G | 0.44/0.11 | 8.15[4.35,15.29] | 6.26×10^-11^ | intergenic,HLA-C(dist=23303),HLA-B(dist=58433) |
| 6 | rs9468933 | 31265057 | A/T | 0.44/0.11 | 8.22[4.37,15.46] | 6.61×10^-11^ | intergenic,HLA-C(dist=25144),HLA-B(dist=56592) |
| 6 | rs13200073 | 31271008 | T/C | 0.44/0.11 | 8.22[4.37,15.46] | 6.61×10^-11^ | intergenic,HLA-C(dist=31095),HLA-B(dist=50641) |
| 6 | rs13216197 | 31271018 | C/T | 0.44/0.11 | 8.22[4.37,15.46] | 6.61×10^-11^ | intergenic,HLA-C(dist=31105),HLA-B(dist=50631) |
| 6 | rs7751729 | 31271936 | T/C | 0.44/0.11 | 8.22[4.37,15.46] | 6.61×10^-11^ | intergenic,HLA-C(dist=32023),HLA-B(dist=49713) |
| 6 | kgp8508902 | 31399821 | T/A | 0.46/0.14 | 7.08[3.91,12.81] | 1.02×10^-10^ | intergenic,MICA(dist=16731),HCP5(dist=31136) |
| 6 | rs9266631 | 31346898 | A/G | 0.51/0.14 | 6.73[3.77,12.01] | 1.07×10^-10^ | intergenic,HLA-B(dist=21909),MICA(dist=24473) |
| 6 | rs9468924 | 31256948 | A/G | 0.37/0.09 | 9.64[4.82,19.26] | 1.41×10^-10^ | intergenic,HLA-C(dist=17035),HLA-B(dist=64701) |
| 6 | kgp11097966 | 31315891 | A/G | 0.52/0.16 | 6.23[3.55,10.95] | 2.01×10^-10^ | intergenic,HLA-C(dist=75978),HLA-B(dist=5758) |
| 6 | kgp3181779 | 31199573 | T/C | 0.38/0.12 | 7.34[3.90,13.81] | 6.79×10^-10^ | intergenic,HCG27(dist=27828),HLA-C(dist=36953) |
| 6 | kgp5593447 | 31326629 | C/T | 0.55/0.19 | 4.80[2.91,7.92] | 7.90×10^-10^ | intergenic,HLA-B(dist=1640),MICA(dist=44742) |
| 6 | kgp8742243 | 31286577 | A/G | 0.45/0.12 | 5.9[3.32,10.49] | 1.51×10^-09^ | intergenic,HLA-C(dist=46664),HLA-B(dist=35072) |
| 6 | rs6457349 | 31203886 | T/A | 0.42/0.15 | 6.15[3.40,11.13] | 1.91×10^-09^ | intergenic,HCG27(dist=32141),HLA-C(dist=32640) |
| 6 | rs7745906 | 31204008 | A/G | 0.42/0.15 | 6.15[3.40,11.13] | 1.91×10^-09^ | intergenic,HCG27(dist=32263),HLA-C(dist=32518) |
| 6 | rs28397299 | 31207704 | A/G | 0.42/0.15 | 6.15[3.40,11.13] | 1.91×10^-09^ | intergenic,HCG27(dist=35959),HLA-C(dist=28822) |
| 6 | rs2244546 | 31435833 | G/C | 0.33/0.09 | 7.74[3.96,15.15] | 2.27×10^-09^ | intergenic,HCP5(dist=2247),HCG26(dist=3173) |
| 6 | rs17198616 | 31247964 | C/T | 0.39/0.14 | 6.31[3.43,11.63] | 3.44×10^-09^ | intergenic,HLA-C(dist=8051),HLA-B(dist=73685) |
| 6 | rs2523627 | 31346445 | C/A | 0.62/0.28 | 4.32[2.66,7.021] | 3.62×10^-09^ | intergenic,HLA-B(dist=21456),MICA(dist=24926) |
| 6 | rs9468932 | 31264823 | A/G | 0.51/0.18 | 4.69[2.80,7.84] | 3.97×10^-09^ | intergenic,HLA-C(dist=24910),HLA-B(dist=56826) |
| 6 | rs9468928 | 31263043 | G/A | 0.52/0.18 | 4.57[2.75,7.60] | 4.47×10^-09^ | intergenic,HLA-C(dist=23130),HLA-B(dist=58606) |
| 6 | rs2894207 | 31263751 | C/T | 0.52/0.18 | 4.57[2.75,7.60] | 4.47×10^-09^ | intergenic,HLA-C(dist=23838),HLA-B(dist=57898) |
| 6 | rs28894987 | 31264302 | C/A | 0.52/0.18 | 4.57[2.75,7.60] | 4.47×10^-09^ | intergenic,HLA-C(dist=24389),HLA-B(dist=57347) |
| 6 | rs2394892 | 31205382 | A/G | 0.40/0.15 | 6.21[3.37,11.43] | 4.68×10^-09^ | intergenic,HCG27(dist=33637),HLA-C(dist=31144) |
| 6 | rs2844552 | 31342741 | C/T | 0.66/0.32 | 3.76[2.40,5.91] | 8.65×10^-09^ | intergenic,HLA-B(dist=17752),MICA(dist=28630) |
| 6 | rs4959062 | 31331500 | C/G | 0.62/0.30 | 4.18[2.56,6.82] | 1.05×10^-08^ | intergenic,HLA-B(dist=6511),MICA(dist=39871) |
| 6 | kgp652609 | 31263051 | G/A | 0.51/0.23 | 4.93[2.84,8.57] | 1.56×10^-08^ | intergenic,HLA-C(dist=23138),HLA-B(dist=58598) |
| 6 | rs9266440 | 31337815 | C/T | 0.34/0.66 | 3.85[2.38,6.25] | 1.75×10^-08^ | intergenic,HLA-B(dist=12826),MICA(dist=33556) |
| 6 | kgp5192277 | 31192998 | A/G | 0.45/0.18 | 4.82[2.79,8.33] | 1.76×10^-08^ | intergenic,HCG27(dist=21253),HLA-C(dist=43528) |
| 6 | rs2442741 | 31346079 | A/G | 0.60/0.28 | 3.71[2.34,5.86] | 2.25×10^-08^ | intergenic,HLA-B(dist=21090),MICA(dist=25292) |
| 6 | kgp10804684 | 31370329 | A/C | 0.51/0.26 | 4.51[2.65,7.66] | 2.53×10^-08^ | intergenic,HLA-B(dist=45340),MICA(dist=1042) |
| 6 | rs2523589 | 31327334 | C/A | 0.64/0.31 | 3.32[2.17,5.06] | 2.69×10^-08^ | intergenic,HLA-B(dist=2345),MICA(dist=44037) |
| 6 | kgp7119073 | 31074829 | T/C | 0.39/0.14 | 4.92[2.81,8.64] | 2.77×10^-08^ | intergenic,HCG22(dist=47176),C6orf15(dist=4171) |
| 6 | rs2442751 | 31351991 | T/C | 0.25/0.56 | 3.85[2.38,6.25] | 3.23×10^-08^ | intergenic,HLA-B(dist=27002),MICA(dist=19380) |
| 6 | rs9266684 | 31348505 | C/T | 0.24/0.55 | 3.85[2.38,6.25] | 3.45×10^-08^ | intergenic,HLA-B(dist=23516),MICA(dist=22866) |
| 6 | rs6927324 | 31194764 | C/A | 0.44/0.18 | 4.64[2.69,8.00] | 3.59×10^-08^ | intergenic,HCG27(dist=23019),HLA-C(dist=41762) |
| 6 | rs2442752 | 31351764 | G/A | 0.49/0.22 | 4.50[2.63,7.72] | 4.52×10^-08^ | intergenic,HLA-B(dist=26775),MICA(dist=19607) |

^#^Results are based on logistic regression models that include principal component 1 as covariate to correct for population stratification. SNPs, positions are based on NCBI build 37. Allele A1 represents minor allele. Odds Ratio was calculated with A2 as the actual reference allele. CHR, chromosome; 95%CI, 95% confidence interval. BP, base position, MAF, minor allele frequency; The SNPs printed in bold are those identified as statistically independent by conditional association study and highlighted in the main text.

### Table S3. Stepwise conditional analysis of association among top 57 SNPs with *P*<5×10^-8^

| Test SNP | *P*_Unconditioned_ | *P*_Conditioned rs2857281_ | *P*_Conditioned rs2857281&rs2523557_ |
| --- | --- | --- | --- |
| rs2523628 | 3.94×10^-14^ | 0.28 | 0.72 |
| rs2857281 | 5.64×10^-14^ | NA | NA |
| rs2844510 | 7.00 | NA | NA |
| kgp1819618 | 2.33×10^-13^ | 0.19 | 0.26 |
| rs2523559 | 2.53×10^-13^ | 9.88×10^-3^ | NA |
| rs2523557 | 2.53×10^-13^ | 9.88×10^-3^ | NA |
| kgp8779347 | 2.66×10^-13^ | 0.83 | 0.76 |
| kgp5542250 | 4.75×10^-13^ | 0.73 | 0.64 |
| rs9500887 | 5.48×10^-13^ | 0.41 | 0.84 |
| kgp11163133 | 1.61×10^-12^ | 0.21 | 0.15 |
| kgp17159519 | 1.76×10^-12^ | 1 | 1 |
| rs9461685 | 3.82×10^-12^ | 0.75 | 0.83 |
| rs9295970 | 3.82×10^-12^ | 0.75 | 0.83 |
| rs10081114 | 3.82×10^-12^ | 0.75 | 0.83 |
| rs9468942 | 3.82×10^-12^ | 0.75 | 0.83 |
| kgp11347311 | 6.11×10^-12^ | 0.05 | 1 |
| kgp8529577 | 7.76×10^-12^ | 0.48 | 0.25 |
| kgp9593289 | 7.76×10^-12^ | 0.48 | 0.25 |
| rs2844586 | 7.83×10^-12^ | 0.88 | 0.69 |
| kgp9149991 | 1.45×10^-11^ | 0.63 | 0.33 |
| rs4143334 | 2.10×10^-11^ | 0.48 | 0.4 |
| rs9468929 | 6.26×10^-11^ | 0.09 | 0.58 |
| rs9468933 | 6.61×10^-11^ | 0.16 | 0.78 |
| rs13200073 | 6.61×10^-11^ | 0.16 | 0.78 |
| rs13216197 | 6.61×10^-11^ | 0.16 | 0.78 |
| rs7751729 | 6.61×10^-11^ | 0.16 | 0.78 |
| kgp8508902 | 1.02×10^-10^ | 0.78 | 0.43 |
| rs9266631 | 1.07×10^-10^ | 0.75 | 0.14 |
| rs9468924 | 1.41×10^-10^ | 0.72 | 0.53 |
| kgp11097966 | 2.01×10^-10^ | 0.4 | 0.73 |
| kgp3181779 | 6.79×10^-10^ | 0.99 | 0.9 |
| kgp5593447 | 7.90×10^-10^ | 0.6 | 0.05 |
| kgp8742243 | 1.51×10^-09^ | 0.64 | 0.23 |
| rs6457349 | 1.91×10^-09^ | 0.89 | 0.77 |
| rs7745906 | 1.91×10^-09^ | 0.89 | 0.77 |
| rs28397299 | 1.91×10^-09^ | 0.89 | 0.77 |
| rs2244546 | 2.27×10^-09^ | 0.17 | 0.22 |
| rs17198616 | 3.44×10^-09^ | 0.3 | 0.31 |
| rs2523627 | 3.62×10^-09^ | 0.78 | 0.15 |
| rs9468932 | 3.97×10^-09^ | 0.26 | 0.87 |
| rs9468928 | 4.47×10^-09^ | 0.2 | 0.99 |
| rs2894207 | 4.47×10^-09^ | 0.2 | 0.99 |
| rs28894987 | 4.47×10^-09^ | 0.2 | 0.99 |
| rs2394892 | 4.68×10^-09^ | 0.98 | 0.92 |
| rs2844552 | 8.65×10^-09^ | 0.82 | 0.27 |
| rs4959062 | 1.05×10^-08^ | 0.76 | 0.06 |
| kgp652609 | 1.56×10^-08^ | 0.05 | 0.17 |
| rs9266440 | 1.75×10^-08^ | 0.57 | 0.91 |
| kgp5192277 | 1.76×10^-08^ | 0.37 | 0.47 |
| rs2442741 | 2.25×10^-08^ | 0.84 | 0.13 |
| kgp10804684 | 2.53×10^-08^ | 0.51 | 0.46 |
| rs2523589 | 2.69×10^-08^ | 0.58 | 0.43 |
| kgp7119073 | 2.77×10^-08^ | 0.34 | 0.56 |
| rs2442751 | 3.23×10^-08^ | 0.05 | 0.39 |
| rs9266684 | 3.45×10^-08^ | 0.04 | 0.35 |
| rs6927324 | 3.59×10^-08^ | 0.35 | 0.43 |
| rs2442752 | 4.52×10^-08^ | 0.54 | 0.88 |
| HLA-B*1301 | 7.52×10^-12^ | 0.004215 | 0.03 |

### Table S4. Evaluation of risk assessment model on discovery and replication dataset

| Stage | AUC (SD) | Sensitivity^1^ (SD) | Specificity^1^ (SD) |
| --- | --- | --- | --- |
| Discovery (10-fold cross validation) | 0.85 (0.07) | 0.78 (0.12) | 0.89 (0.13) |
| Replication study | 0.82 | 0.74 | 0.85 |

^1^ sensitivity and specificity were calculated with default cutoff of zero point

AUC, area under receiver operating characteristic curve. SD, standard deviation.

Table S5. Power calculation

| Risk Allele Frequency | Genotype Relative Risk (Additive Model) | | | | | | | | | |
| --- | --- | --- | --- | --- | --- | --- | --- | --- | --- | --- |
|  | 1.20 | 1.30 | 1.40 | 1.50 | 1.60 | 1.70 | 1.80 | 2.00 | 5.00 | 10.00 |
| 0.04 | 1.41E-07 | 2.81E-07 | 8.85E-07 | 1.33E-06 | 1.47E-07 | 5.11E-06 | 8.48E-06 | 2.60E-05 | 0.081 | 0.842 |
| 0.05 | 1.90E-07 | 3.99E-07 | 8.50E-07 | 2.48E-06 | 4.23E-06 | 7.95E-06 | 1.51E-05 | 5.06E-05 | 0.159 | 0.936 |
| 0.06 | 2.66E-07 | 5.79E-07 | 1.25E-06 | 2.74E-06 | 5.94E-06 | 1.35E-05 | 2.58E-05 | 8.97E-05 | 0.255 | 0.974 |
| 0.07 | 3.84E-07 | 8.57E-07 | 1.83E-06 | 4.03E-06 | 8.91E-06 | 1.92E-05 | 3.97E-05 | 1.49E-04 | 0.355 | 0.988 |
| 0.08 | 2.38E-07 | 6.98E-07 | 1.93E-06 | 5.87E-06 | 1.30E-05 | 2.84E-05 | 5.98E-05 | 4.10E-04 | 0.451 | 0.994 |
| 0.09 | <0.001 | <0.001 | <0.001 | <0.001 | <0.001 | <0.001 | <0.001 | 3.39E-04 | 0.537 | 0.997 |
| 0.10 | <0.001 | <0.001 | <0.001 | <0.001 | <0.001 | <0.001 | <0.001 | 4.80E-04 | 0.610 | 0.998 |
| 0.15 | <0.001 | <0.001 | <0.001 | <0.001 | <0.001 | <0.001 | <0.001 | 1.73E-03 | 0.823 | 0.9996 |
| 0.20 | <0.001 | <0.001 | <0.001 | <0.001 | <0.001 | <0.001 | <0.001 | 3.86E-03 | 0.897 | 0.9998 |

| CHR | SNP | POS | Allele frequency in control group | Odds Ratio^#^ | POWER | N cases for  80% power |
| --- | --- | --- | --- | --- | --- | --- |
| 6 | rs2857281 | 31374262 | 0.056 | 24.21 | 100% | 43 |
| 6 | rs2523557 | 31331257 | 0.107 | 11.22 | 99.88% | 56 |

Top panel: Study power as a function of disease allele frequency and genotype relative risk. Bottom panel: Power of two independent SNPs. These power calculations are based on discovery dataset. ^#^Odds Ratio was estimated by our study. Assuming alpha=5×10^-8^, sample size = 196 (with 98 cases and 98 controls), prevalence = 0.01.
